# The role of complement and extracellular vesicles in the development of pulmonary embolism in severe COVID-19 cases

**DOI:** 10.1371/journal.pone.0309112

**Published:** 2024-08-23

**Authors:** Gabriel Dumitrescu, Jovan Antovic, Nida Soutari, Charlotte Gran, Aleksandra Antovic, Kais Al-Abani, Jonathan Grip, Olav Rooyackers, Apostolos Taxiarchis

**Affiliations:** 1 Division of Anaesthesia and Intensive Care, Department of Clinical Science, Intervention and Technology, Karolinska Institutet, and Perioperative and Intensive Care Medicine, Karolinska University Hospital, Stockholm, Sweden; 2 Department of Molecular Medicine and Surgery, Karolinska Institutet, and Clinical Chemistry, Karolinska University Laboratory, Karolinska University Hospital, Stockholm, Sweden; 3 Division of Rheumatology, Department of Medicine, Karolinska Institutet, and Unit of Rheumatology, Karolinska University Hospital, Stockholm, Sweden; 4 Department of Clinical Physiology, Karolinska University Hospital, Stockholm, Sweden; Nagoya University Graduate School of Medicine, JAPAN

## Abstract

Complement and extracellular vesicles (EVs) association with thrombogenic tendencies is acknowledged, but limited evidence exists for their link to COVID-19 venous thromboembolism. This study aims to examine the relationship between pulmonary embolism and the expression of complement and other proteins related to thrombogenesis in severe Covid-19 patients. We included prospectively 207 severe COVID-19 patients and retrospectively screened for pulmonary embolism (PE). This analysis comprises 20 confirmed PE cases and 20 matched patients without PE. Blood samples taken at the admission in the intensive care unit were analyzed for complement using ELISA. EVs derived from neutrophils, endothelium, or platelets, as well carrying complement or tissue factor were analyzed using flow cytometry. Complement levels were markedly elevated, with a notable increase in C3a and Terminal Complement Complex. The most prevalent EV population was identified as tissue factor (TF)-carrying EVs which peaked in patients with PE during ICU days 4–9. However, for both the complement and analyzed EV populations, no statistically significant differences were found between the patients who developed pulmonary embolism and those who did not. In conclusion, complement factors and EVs expressing tissue factor, along with EVs derived from endothelial cells and platelets, are elevated in severe COVID-19 patients, regardless of the presence of pulmonary embolism. However, the involvement of complement and procoagulant EVs in peripheral plasma in the development of pulmonary embolism is still unclear and requires further investigation.

## Introduction

The Covid-19 virus, having culminated in a global pandemic, has impacted millions of people worldwide. One of the most concerning aspects of this disease characterized by a hyper-inflammatory response [1] is its propensity to hypercoagulability [2] which significantly elevates the risk for pulmonary embolism (PE) [3] and, consequently, raises mortality rates [4, 5].

The complement system, as an integral component of the immune system, plays a crucial role in the host’s defence against various pathogens, including viruses. Disruption in its regulation can substantially contribute to the development of inflammatory-mediated pathologies [6]. Therefore, while it is anticipated that the complement system would offer protection against COVID-19 infection, a growing body of evidence suggests its involvement in the development of the disease [7–11]. Within this context, the thrombogenic tendency associated with COVID-19 may hypothetically originate from complement dysfunction [12], given the bidirectional communication and interaction between the complement and the coagulation system [13, 14]. However, the current evidence is not sufficient to reach a conclusion regarding the value of the complement system as biomarker or as potential cause of COVID-related venous thromboembolism (VTE).

Extracellular vesicles (EVs), also known as microparticles or microvesicles, are membrane vesicles ranging in size from 0.03 to 1 μm in diameter and they originate from various cellular sources. They are actively shed from cells in response to various triggers such as activation, injury, and apoptosis, mediating intercellular communication and carrying crucial biological information [15–17]. EVs have been regarded as potential biomarkers for assessing endothelial dysfunction, vascular injury and identifying pro-thrombotic or pro-inflammatory states [16, 18], nevertheless, their involvement in intercellular communication also raises questions regarding their potential role in the pathogenesis of diseases [17]. Several recent studies have shown that Covid-19 is associated with increased numbers of circulating EVs [19–21]. However, there is limited evidence regarding their relationship with VTE in Covid-19.

The aim of this study was to explore the interplay between development of pulmonary embolism and expression of complement and other thrombogenesis-related proteins in patients suffering from severe Covid-19 infection. Hence, our objective was to investigate and quantify the total plasma levels of complement proteins, along with the levels of complement and tissue factor (TF) expression on the surface of EVs derived from different cellular origin, with the aim of identifying potential markers for the risk of PE.

## Materials and methods

### Ethics

The protocol was approved by the Swedish Ethical Review Authority (Dnr 2020–01302) and was in accordance with the Declaration of Helsinki of 1975 and its subsequent revisions. Informed consent was obtained from the patient when possible. In unconscious patients, consent was obtained within 90 days of ICU admission or, if the patient died, was assumed.

### Patients

In this retrospective analysis of a prospective single-center study, we included 207 patients admitted from October 2020 until May 2021 in the intensive care unit (ICU) at Karolinska University Hospital, Stockholm, Sweden, due to severe Covid-19 pneumonia (severe respiratory failure with PaO2/FiO2 < 20 kPa, respiratory rate > 30/min, indication for mechanical ventilation).

The patients were systematically screened with computed tomography pulmonary angiography (CTPA) for PE during the entire ICU stay according to hospital protocols. This involved performing the scan at admission, within the first 3 days, then twice more at intervals of 5 days, and additionally whenever clinical or laboratory signs of possible PE appeared. All the patients received VTE prophylaxis according to the hospital routines with Fragmin, typically administered at a dose of 5000 IU twice daily.

PE was identified in 20 patients, constituting 10% of the cohort. The diagnosis occurred between ICU days 0–20. Seventeen of the patients had segmental PE whereas three had central PE. These PE patients did not have risk factors associated with VTE, except for prolonged bedridden immobility.

Using the included patients, we constructed a matching control group (n = 20) according to age, weight, gender and the Charlson comorbidity index corrected for age from the rest of the cohort. The patient’s and matching control group characteristics are given in Table 1.

**Table 1:** Patients’ characteristics. Biochemical and coagulation tests.

		No PE(n = 20)	PE(n = 20)	*p*
**Older (years)**		67(8)*	68(8)*	0.64
**Weight (kg)**		81(10)*	76(16)*	0.25
**Gender (nr.)**		14 M /6 W	15 M / 5 W	-
**Charlson index corrected for older**		3[1–7]	3.5 [1–10]	0.55
	**Reference interval**			
**CRP**	< 3 mg/L	130[2–298]	142[37–435]	0.12
**Procalcitonin**	<0.5 μg/L	0.42[0.12–9.6]	0.54[0.06–6.2]	0.78
**IL6**	<7ng/L	93[7.6–244]	244[32–2396]	0.12
**Ferritin**	30–350 μg/L	1300[192–4858]	1433[281–4561]	0.57
**D Dimer**	<0,74 mg/L	1.6[0.2–10.4]	2.2[0.47–35]	0.33
**Fibrinogen**	2–4.2 g/L	5.7(1.5)*	5.8(2.0)*	0.90
**APT**	20–30 s	24[20–38]	26[20–46]	0.11
**INR**	<1.2	1.1[1–2.8]	1.1[0.9–4.3]	0.37
**Platelets**	145–348 x10(9)/L	225[81–574]	203[81–638]	0.35

The values are given as median [range] or mean (SD)*, (No PE = patients without PE, PE = patients with PE; M = men, W = women)

*Standard deviation

### Plasma sampling

Blood samples were obtained within 24 hours from admission to the ICU. Blood was drawn from the existing arterial line into Vacutainer Tubes containing K2 EDTA (BD Vacutainer® PPT™). Within one hour of collection, the tube was subsequently centrifuged at 3000 × *g* for 15 min at 4°C for preparation of platelet-poor plasma. Tubes were immediately frozen and stored at −80°C until further analysis.

### Flow cytometric analysis of EVs

Frozen plasma samples were thawed for 4 min at 37°C and then immediately processed for the evaluation of EVs of size 0.1–1 μm, as described by Taxiarchis et al. [22], using a FACs Canto flow cytometer (BD Biosciences). 20 μl of plasma were incubated with antibodies for 30 min at RT in the dark. To stop the incubation, 500 μl of sterilized PBS was added. For EV size calibration of the flow cytometer and determination of the EV gate, we used Megamix-Plus SSC beads (BioCytex) of sizes 0.16, 0.20, 0.24 and 0.5 μm on side scatter. List of antibodies and their concentration can be found in S1 Table. Annexin V was used as a positive marker of EVs.

The fluorescent antibody panels were the following: (a) Annexin V-PerCP-Cy5.5, anti-TCC-FITC, anti-MASP-2-PE, MPO-APC; (b) AnnexinV-PerCP-Cy5.5, anti-C3a-FITC, anti-CFD-PE, MPO-APC; (c) AnnexinV-PerCP-Cy5.5, anti-CD144-FITC, anti-CD54-PE, anti-CD62P-APC, anti-CD62E-PE-Vio770; (d) AnnexinV-PerCP-Cy5.5, anti-CD142-APC. The selection of populations is presented in S1 Fig. To minimize the effects of spectral overlap of fluorochromes and to define the gating strategy, Fluorescence minus one (FMO) staining was performed for each antibody (S2 Fig). Results are expressed as EV events/μL, using FACS Canto volume measurement over 90 sec. Data files were exported and evaluated by FlowJo software (BD Biosciences).

### ELISA

Evaluation of complement concentrations in plasma was performed in freshly thawed samples with the following ELISA kits according to their instructions: Human Terminal Complement Complex (TCC) HK328 (Hycult Biotech), MASP-2 HK326 (Hycult Biotech), Human C3a ELISA Kit (Invitrogen), Human Complement Factor D Quantikine ELISA Kit (R&D Systems).

### Statistics

Data was analyzed by using Graph Pad Prism (version 9.1 for Windows) and SPSS Statistics (26 IBM). We assessed the normality of the data using the Shapiro-Wilk test. The results are presented either as mean ± standard deviation of the mean for parametric data or median and range for nonparametric data. Differences in estimated variables between groups were evaluated using Student’s t-test for parametric data and the Mann-Whitney unpaired t-test for nonparametric data. The Kruskal-Wallis test was used for multiple comparisons. The Spearman correlation test was employed to assess correlations. Statistical significance was defined as p ≤ 0.05.

## Results

### Coagulation and biochemical analyses

Table 1 provides the routine biochemistry analysis for coagulation and inflammation markers. APT, INR, and platelet levels were mostly within the reference range in both groups. While fibrinogen and D-dimer values exceeded normal ranges in both groups, there was no statistically significant difference between them. Biochemical tests for inflammation, including CRP, IL6, and ferritin, showed levels largely above the normal range in both groups, with no statistically significant difference between the two groups.

### Complement factor levels in plasma

Table 2 presents the results for the analyzed complement factors in plasma. MASP2, C3a, and TCC levels were notably elevated, particularly for TCC, surpassing the normal ranges. On the other hand, CFD exhibited values that, on average (median), remained within the reference interval. No statistically significant differences were observed between the two groups in any of the analyzed markers.

**Table 2:** Complement concentration in plasma.

	Reference interval	No PE(n = 20)	PE(n = 20)	*p*
**MASP2**	170 to 1196 ng/ml	1807(725)*	2179(1057)*	0.20
**CFD**	2297 (2198)* ng/ml	1476[916–6280]	1594[912–2820]	0.68
**C3a**	50–200 ng/ml	1357(664)*	1469(569)*	0.48
**TCC**	< 1000 mAU/ml	4330[2200–7430]	4520[2028–7640]	0.93

The values are given as median [range] or mean (SD)*. (No PE = patients without PE, PE = patients with PE, AU = arbitrary units)

*Standard deviation

### Expression of extracellular vesicles

The concentration of EVs is presented in Table 3. Most abundant EV population was TF carrying EVs (TF+EVs)/ (CD142+). There were no statistically significant differences between the two patient groups for any of the analyzed EV populations, whether derived from neutrophils (MPO+) or endothelium (CD144+ and CD54+), and whether carrying complement (MASP2+, CFD+, C3a+, TCC+, MASP2+ MPO+, CFD+ MPO+, C3a+ MPO+, TCC+ MPO+) or tissue factor (CD142+), or bearing activation markers for endothelium (CD62-E+) or platelets (CD62-P+). Graphic presentation of results is provided in the S3 Fig.

**Table 3:** Extracellular vesicles in plasma (events/μL).

	No PE(n = 20)	PE(n = 20)	p
*Complement carrying EVs*			
**MASP2 +**	45[15–145]	47 [14–75]	0.64
**CFD+**	140[51–676]	184 [60–407]	0.41
**C3a+**	2332[605–12029]	2744 [548–11619]	0.39
**TCC+**	1848[343–9475]	1953 [870–5194]	0.94
*TF carrying EVs*			
**CD142+**	13701[2405–140016]	22369[2605–90234]	0.26
*Neutrophil-derived EVs*			
**MPO+**	331[49–2000]	382[85–1123]	0.67
*Complement carrying neutrophil-derived EVs*			
**MASP2+ MPO+**	20 [8–106]	22 [3–53]	0.95
**CFD+ MPO+**	23 [9–136]	25 [9–69]	0.78
**C3a+ MPO+**	123 [9–730]	146 [17–319]	0.82
**TCC+ MPO+**	397 [79–1675]	293 [81–375]	0.31
*Endothelium-derived EVs*			
**CD144+**	70 [4–2298]	129 [3–954]	0.62
**CD54+**	391 [88–1974]	392 [8–1212]	0.53
**CD62-E+**	40 [21–65]	37 [11–61]	0.79
*Platelets-derived EVs*			
**CD62-P+**	1032 [229–2076]	749 [21–3093]	0.10

The values are given as median [range]. (No PE = patients without PE, PE = patients with PE)

Because in average the levels of TF+EVs in plasma were higher in patients with PE compared to the matched control group, in a post-hoc analysis we excluded the two outliers with significantly higher levels of TF+EVs in the control group. The t-test demonstrated an improvement from p = 0.26 to p = 0.07, yet it did not reach statistical significance (S4 Fig).

### Correlation of coagulation tests with EVs

Significant correlations were found between the circulating levels of EVs and coagulation markers across the entire patient cohort (Table 4). D-dimer was significantly correlated with endothelial-derived EVs (CD62E+) whereas fibrinogen was significantly correlated with TF+EVs (CD142+), neutrophil-derived EVs (MPO+) and endothelial-derived EVs (CD162E). INR was significantly correlated with C3a+, TCC+, TF+, MPO+ and endothelial-derived EVs (CD162E and CD54) together with platelet derived EVs (CD62-P+). Platelets were significantly correlated with C3a+, TF+, MPO+, C3a+MPO+, CD54+ and CD62P+ EVs. The Spearman’s correlation coefficients are given in Table 4.

**Table 4:** The Spearman’s correlation coefficients of extracellular vesicles with coagulation biomarkers in COVID-19 patients with and without PE (n = 40).

	D Dimer	Fibrinogen	INR	Platelets
*Complement-carrying EVs*				
**MASP2 +**	0.12	0.05	-0.15	-0.08
**CFD+**	0.10	0.19	-0.18	0.27
**C3a+**	0.04	0.26	**-0.35** ^*^	**0.34** ^*^
**TCC+**	-0.13	0.13	**-0.32** ^*^	0.13
*TF-carrying EVs*				
**CD142+**	0.10	**0.34** ^*^	**-0.49** ^**^	**0.40** ^*^
*Neutrophil-derived EVs*				
**MPO+**	0.15	**0.39** ^*^	**-0.34** ^*^	**0.49** ^**^
*Complement-carrying neutrophil-derived EVs*				
**MASP2+ MPO+**	0.23	0.02	-0.04	-0.19
**CFD+ MPO+**	0.24	-0.03	0.18	0.08
**C3a+ MPO+**	0.09	0.17	-0.22	**0.42** ^**^
**TCC+ MPO+**	-0.14	0.17	-0.19	0.16
*Endothelium-derived EVs*				
**CD144+**	0.02	0.20	**-0.34** ^*^	0.20
**CD54+**	0.17	0.30	**-0.35** ^*^	**0.41** ^**^
**CD62-E+**	**0.38** ^*^	**-0.40** ^*^	0.17	-0.08
*Platelets-derived EVs*				
**CD62-P+**	0.07	0.15	**-0.35** ^*^	**0.50** ^**^

Statistically significant correlations are highlighted in bold in the table.

* p < 0.05

** p < 0.01

### Post hoc analysis

**Fig 1:**
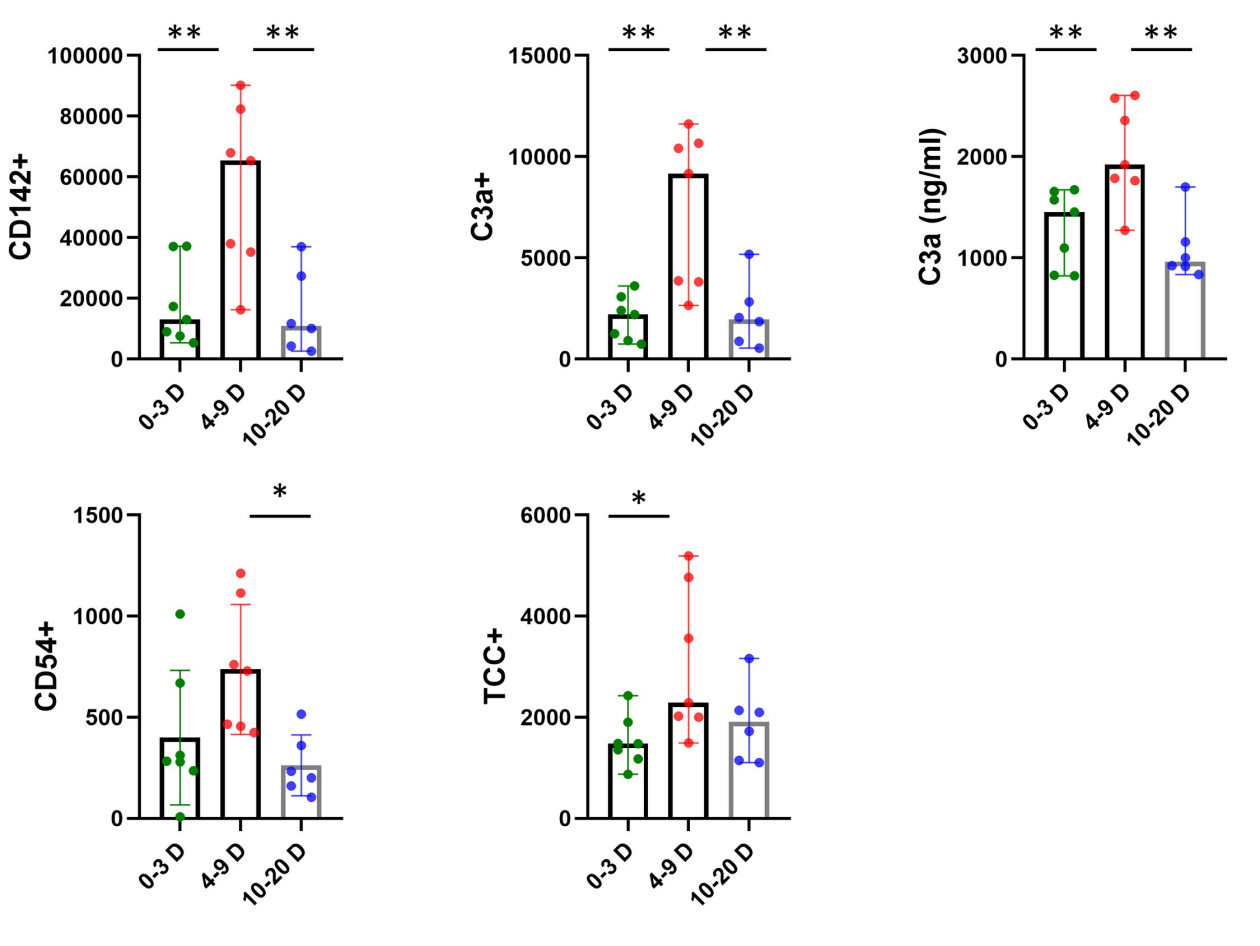
Significant findings in patient subgroups related to the occurrence of PE: Early (0–3 days), interim (4–9 days), and late (10–20 days). The outcomes are presented in median-range format. Level of significance *p = 0.005–0.05; **p<0.005. (D = day of ICU stay).

Due to the variation in timing between sampling and PE diagnosis, we categorized patients with PE into three subgroups based on the time elapsed between sampling and PE detection: early (0–3 days), interim (4–9 days), and late (10–20 days) (Fig 1). In the interim group the levels of TF+EVs (CD142+) were significantly higher compared to the early and late groups. TF+EVs levels in the interim group were also significantly higher compared to the patients without PE (S5 Fig). In the interim group, for complement-carrying EVs, C3a+ were significantly elevated compared to the early and late groups, reflecting the concentration of C3a in plasma which was higher in these patients (Fig 1). Additionally, TCC+ EVs were also significantly higher in the interim group when compared to the early group. EVs originating from activated endothelium (CD54+) were also more abundant in the interim group compared to the late group.

## Discussions

In this retrospective study we investigated whether plasma levels of complement and/or EVs displaying on surface complement or other proteins involved in thrombogenesis, assessed upon admission to the ICU in patients with severe Covid-19 infection, are associated with the development of PE and could therefore serve as potential markers of PE risk.

It is widely acknowledged that the process described as immunothrombosis, is responsible for the microthrombi observed in the lung capillaries and other microvascular areas in COVID-19 patients [23, 24]. Nevertheless, the precise contributions of neutrophils, neutrophil extracellular traps (NETs), activated platelets, extracellular vesicles, clotting factors, inflammatory cytokines, and complement in immunothrombosis on micro- and macro scale continue to be uncertain in various aspects [25].

PE incidence among COVID-19 patients can vary between 7.8%-16.5% [3, 26]. Even with effective antithrombotic drugs, thrombotic events cannot be completely prevented [27, 28]. This suggests that there are uncharted mechanisms at play in the pathogenesis of LE in COVID-19.

The potential involvement of the complement system in the intricate pathophysiology of immunothrombosis in COVID-19 is highlighted by several studies [10, 29–32]. Of note, deposits of Mannan-Binding Lectin Serine Protease 2 (MASP2), C4a, C3, and terminal complement complex (TCC), also referred to as C5b‐9 or Membrane Attack Complex (MAC) are identified in biopsies of lung and skin tissue with microvascular thrombosis highlighting a potential contribution to activation of the coagulation [31, 32]. In addition, specific complement proteins have garnered research interest as potential indicators of disease severity and outcome. Complement components, such as C4d, C3a, and TCC, are elevated in COVID-19 patients in the intensive care unit compared to normal levels, underscoring their potential as markers for disease severity and outcome [33].

In our study, we explored the initiation of complement activation by examining MASP2 (for the lectin pathway) and factor D (for the alternative pathway). As a midway global indicator of activation, we chose to analyse C3a. Ultimately, we investigated the presence of the TCC. Prior findings influenced our decision not to include other fractions such as C4a, C4d, C5a, and complement factor H (CHF). C4a lacks a receptor and is not found on EVs [34], our previous study showed no difference in C4d expression between severe COVID-19 patients and controls [22], and we prioritized C3a over C5a for its subtler role in inflammation [35]. CFH was not included because its role as a negative regulator is affected by competition with the SARS-CoV-2 spike protein [7].

In our subjects, including only ICU patients, the degree of complement activation was highly elevated, especially regarding C3a and TCC, even in comparison to findings in severely ill patients from other studies [33, 36]. Thus, our study corroborates earlier findings, indicating a heightened activation of the complement system in these patients. MASP2 and TCC have been previously demonstrated as activators of thrombin generation [8], as well C3a is reported to have the ability to enhance coagulation through the binding to the C3a receptor (C3aR) located on platelets [7]. However, we found no significant differences between patients with and without PE. This suggests that, in terms of the risk of PE in our cohort, the influence of complement activation may not have been decisive. This implies that, while patients with COVID-19 typically exhibit a higher prevalence of VTE [3], and complement plays a role in the pathophysiology of immunothrombosis, especially concerning the microcirculation [30, 31], complement activation is not alone sufficient to define the risk for PE.

Pro-inflammatory stimuli have been identified as key factors responsible for EVs formation [16, 37, 38]. This interplay between inflammation and EVs emphasizes their procoagulant activities and relevance in conditions such as thrombosis [17]. In a previous study, we demonstrated the presence of TCC and C3a complement proteins on the EV surface. We showed that TCC+ EVs are found in significantly higher numbers in COVID-19 patients compared to healthy controls. Similarly, levels of C3a expression on neutrophil derived EVs (C3a+ MPO+) are higher [22]. Moreover, neutrophil derived EVs carrying tissue factor (TF+ MPO+) have been shown to be elevated in COVID-19 patients [39] as well as in inflammatory conditions, such as anti-neutrophil cytoplasm antibody (ANCA)-associated vasculitis (AAV), and they are associated with increased thrombin generation [40]. For this, our study investigated the expression of complement and tissue factor exposed on the surface of total EVs, along with complement specifically expressed on neutrophil-derived EVs (MPO+ EVs). Additionally, we examined EVs originating from endothelial cells (CD144+ EVs), as well as from activated platelets (CD62-P+ EVs) and activated endothelium (CD62E+ EVs, CD54+ EVs).

We found that the levels of complement proteins expressed on EVs (total EVs or MPO+ EVs), similarly to the concentration of plasma complement proteins, were not significantly different in patients who developed PE compared to those without PE. Additionally, levels of EV populations expressing C3a complement (both on total and MPO+ populations) showed a moderate association with coagulation markers such as INR and platelets, which might suggest a potential role in the activation of coagulation. However, based on our findings, we conclude that although complement proteins together with MPO+ neutrophils are present in high levels in severe Covid-19 patients [22], potentially causing pulmonary deposition of MPO+ neutrophils, MASP-2, CFD and C3d as demonstrated by *Niederreiter et al*. [41], they may not signal the risk of PE. Certainly, the over activation of the innate immune response can precipitate respiratory failure [33, 41]; however, this does not seem to be directly linked to PE in our cohort.

The procoagulant activity of EVs is notably heightened by the presence of tissue-factor. The importance of EVs carrying tissue factor (TF+EVs) was described in two previous studies where no notable distinctions were observed among EVs subsets between moderate and severe forms of COVID-19 [20], except for TF+EVs; the activity of TF expressed on EVs is significantly higher in patients with severe disease when compared to those with a moderate form of the illness [19, 20]. In our cohort, TF+EVs overwhelmingly dominated the overall landscape of EVs from a numerical standpoint. This leads us to believe that they were present in a significantly higher quantity than usual in our patients, all of whom were in a critical stage of the disease. Moreover, as expected, levels of TF+EVs, similar to neutrophil-derived (MPO+) EVs, showed a significant positive correlation with INR, fibrinogen and platelets count. Essential to mention, *Guervilly et al*. noted a significant increase in TF+EVs activity among patients who encountered thromboembolic events (TEEs), including eight cases of deep vein thromboses (DVTs), two cases of PE, and one ischemic stroke. However, in that study, although TF+EVs activities are higher in patients with TEE compared to the entire cohort, which include moderate cases, they do not differ when compared to the severe cases subgroup [20].

We observed in our cohort including exclusively severe cases, that there was no significant difference in TF+EVs between the group that developed PE compared to the control group. Even after exclusion of two out-layers from the control group the observed difference did not reach a statistically significant level (p = 0.07) (S4 Fig).

Noticeable, in the subgroup where PE occurred between days 4 and 9 of ICU stay, the levels of TF+EVs were significantly higher compared to both the patients who developed PE early (ICU days 0–3) or late (ICU days 10–20). We can speculate that, in this particular subgroup, patients were likely in the phase of DVT development, which could be related to the higher levels of TF-EVs as suggested by *Guervilly et al*. and others [20, 42]. The occurrence of PE between days 4–9, may have stemmed from a matured DVT. In contrast, for the early and late groups, the development of DVT might have occurred either before or after sampling, potentially accounting for the comparatively lower levels of TF+EVs. We did not screen the patients for DVT to establish a connection with PE in our cohort. However, previous studies indicate that in Covid-19 pneumonia, the origin of PE is often not associated with DVT [43], as DVT is absent in the majority of Covid-19 patients with PE [44]. Instead, in these patients, *in situ* pulmonary thrombosis may occur due to hypercoagulability induced by systemic inflammation [44, 45]. Additionally, in the subgroup with PE occurring between days 4 and 9 (interim), similar to TF+EVs, C3a+ EVs were significantly higher compared to both early and late groups, aligning with C3a concentration, as well TCC+EVs and activated endothelium derived EVs (CD54+) showed elevated levels. This observation is consistent with the triad of hyperinflammation, endothelial activation, and a procoagulant state which theoretically could determine, if the necessary levels are reached, the formation of *in situ* pulmonary thrombi [45–47].

However, aside from these hypotheses, we infer that the plasma levels of TF-EVs at ICU admission did not conclusively contribute to the onset of PE, nor could they serve as a signalling marker for the risk of PE in patients with severe COVID-19 pneumonia. Yet, the timing of sampling is essential, as the levels of EVs may be higher specifically during thrombus formation, possibly reaching a peak during thrombus development, and then decreasing during PE occurrence. Given that the pulmonary epithelium is acknowledged as a primary source of tissue factor in the lungs [19], it is plausible that under severe Covid-19 pneumonia, as observed in our patients, the pneumocytes could emerge as the primary source of TF-EVs. This could be the explanation for why TF-EVs are not elevated in COVID-19 patients without severe pulmonary disease [20, 22].

During the course of severe Covid-19 infection, widespread inflammation characterized by significantly increased expression of proinflammatory cytokines, such as TF, is the leading cause of endothelial dysfunction [48]. The coagulation potential of dysfunctional endothelium is manifested through platelet stimulation and accelerated thrombotic potential. Together with platelets, dysfunctional endothelial cells play a significant role in immunothrombosis. In these patients, platelet activation is evidenced by elevated P-selectin (CD62P) expression [49]) whereas endothelial activation is observed by elevated E-selectin (CD62E) expression together with endothelial-derived EVs (CD144+ EVs) [50]. In our study groups, while platelet activation was apparent through elevated levels of P-selectin-carrying EVs, which also correlated with INR and platelet count, there was no significant difference between patients who developed LE compared to those who did not. Similarly, no statistically significant difference in the expression of endothelial EVs or CD62E+ EVs. Worth to mention, in *Mezine et al* [50] the difference was demonstrated between critically ill and non-critically ill patients, without taking PE into account. Our study demonstrates that the development of PE is not directly related to the levels of platelet or endothelial activation.

The pathogenesis of VTE is multifactorial. Virchow’s Triad, which remains relevant, identifies three key factors contributing to thrombosis formation: venous stasis, vascular injury, and hypercoagulability. Among these factors, reduced blood flow is considered the most significant, despite that alone is not sufficient to trigger thrombus formation [51]. Given that our study did not identify the complement and/or procoagulant EVs as indisputable contributing factors, it appears to support this hypothesis for Covid-19 severe infection.

The strengths of our study include: i) strict inclusion of patients with severe cases of COVID-19, ii) a relatively large number of screened subjects, with a sufficiently high number of diagnosed PE events, iii) we were able to very well matched groups, iv) utilization of flow cytometry for the assessment of EVs. A limitation of this study is its retrospective nature, which resulted in a lack of control over the timing of sampling in relation to pulmonary PE events.

## Conclusions

Our study confirmed complement activation and elevated levels of EVs in patients with severe COVID-19, yet these biological markers did not indicate an increased risk of PE at the time of admission in the ICU through their concentrations in peripheral plasma. However, our findings suggest that the pathogenesis of pulmonary embolism in severe COVID-19 cases may potentially be linked to deep vein thrombosis, where tissue factor-carrying extracellular vesicles might play a role, as well that further prospective research is needed to clarify the involvement of complement and procoagulant EVs during the venous thromboembolism process.

## Supporting information

S1 Table List of antibodies. (PDF)

S1 Fig Flowcytometry setup and gating strategy for characterization of EV subpopulations. (a) Calibration for determination of EVs in the FITC vs. SSC dot plot by using 0.16–0.5 μm standard Megamix SSC-Plus beads; Approx. 90% of the counts in the gate were detected within the EV size-gate, as described in Taxiarchis et al, 2023. Events positive to Annexin V were considered as EVs. Process of choosing the subpopulations of: (b) MASP2+, TCC+, MPO+, TCC+MPO+, MASP2+MPO+ EVs. (c) CFD+, C3a+, CFD+MPO+, C3a+MPO+ EVs. (d) endothelial-derived EVs (CD144+ and CD54+), activated platelet-derived EVs (CD62P+) and activated endothelial-derived EVs (CD62E+) and (e) tissue factor (CD142+).
(PDF)

S2 Fig Fluorescence minus one (FMO) control. To identify all our sub-populations, all gates were controlled in pooled plasma derived from the patients using a stain that lacks just one of the fluorescent markers of interest.
(PDF)

S3 Fig Extracellular vesicles (EVs). The outcomes are given as events/μL. The Mann-Whitney test was used for comparisons, and the corresponding p-value is displayed in the upper right corner of the image. (no PE = no pulmonary embolism subgroup; PE = pulmonary embolism subgroup).
(PDF)

S4 Fig Tissue factor carrying extracellular vesicles (CD 142+) in all patients (panel A) and in all patients’ whiteout 2 outstanding outliers in the subgroup without pulmonary embolism (Panel B). The outcomes are given as events/μL. The Mann-Whitney test was used for comparisons, and the corresponding p-value is displayed in the upper right corner of the image. (no PE = no pulmonary embolism subgroup; PE = pulmonary embolism subgroup).
(PDF)

S5 Fig Tissue factor carrying extracellular vesicles (CD 142+) in patient subgroups related to the occurrence of PE: early (0–3 days), interim (4–9 days), and late (10–20 days), and in the subgroup without pulmonary embolism including (panel A) and excluding (panel B) two outstanding outliers. The outcomes are given as events/μL The Mann-Whitney test was used to compare the interim subgroup (4–9 days) with the subgroup without pulmonary embolism, and the corresponding p-value is displayed on the top of the image. (D = day of ICU stay; no PE = no pulmonary embolism subgroup)
(TIF)

S1 Data Minimal data set including patients’ results and correlations reported in the article. (XLSX)
