# Supplementary material for: The role of complement and extracellular vesicles in the development of pulmonary embolism in severe COVID-19 cases
Source: PLoS One. 2024 Aug 23;19(8):e0309112. doi: 10.1371/journal.pone.0309112 (PMC11343408; doi:10.1371/journal.pone.0309112)
Supplement: S1 Table — (PDF) [file pone.0309112.s001.pdf]

S1 Table. List of Antibodies

Antibodies	Source	Identifier	Concentration	Dilutions
**PE anti-human MASP-2 (B-10)**	Santa Cruz Biotechnology	sc-390200 PE	200 µg/ml	1:100
**PE anti-human CFD (Adipsin) (D10/4)**	Santa Cruz Biotechnology	sc-47683 PE	200 µg/ml	1:100
**FITCH anti-human C3a (K13/16-5.7)**	Novus Biologicalis	NBP1-05122F	1000 µg/ml	2:100
**FITCH anti-human TCC (aE11)**	USBiological	298100	1000 µg/mL	2:100
**APC anti-human Myeloperoxidase (REA491)**	Miltenyi	130-119-786	2 µl/test	
**PE-Vio 770 anti-human CD62E (REA280)**	Miltenyi	130-106-591	5 µl/test	
**APC anti-human CD142 (HTF-1)**	Miltenyi	130-098-744	5 µl/test	
**APC anti-human CD62P (AK-4)**	BD Biosciences	550888	5 µl/test	
**PerCP-Cy5.5 anti-human Annexin V**	BD Biosciences	561431	5 µl/test	
**FITC anti-human CD144 (55-7H1)**	BD Biosciences	560411	5 µl/test	
**PE anti-human Myeloperoxidase (5B8)**	BD Biosciences	333139	5 µl/test	
**PE anti-human CD54 (HA58)**	Invitrogen	12-0549-42	5 µl/test	
