# Supplementary material for: The role of complement and extracellular vesicles in the development of pulmonary embolism in severe COVID-19 cases
Source: PLoS One. 2024 Aug 23;19(8):e0309112. doi: 10.1371/journal.pone.0309112 (PMC11343408; doi:10.1371/journal.pone.0309112)
Supplement: S1 Fig — (a) Calibration for determination of EVs in the FITC vs. SSC dot plot by using 0.16–0.5 μm standard Megamix SSC-Plus beads; Approx. 90% of the counts in the gate were detected within the EV size-gate, as described in Taxiarchis et al, 2023. Events positive to Annexin V were considered as EVs. Process of choosing the subpopulations of: (b) MASP2+, TCC+, MPO+, TCC+MPO+, MASP2+MPO+ EVs. (c) CFD+, C3a+, CFD+MPO+, C3a+MPO+ EVs. (d) endothelial-derived EVs (CD144+ and CD54+), activated platelet-derived EVs (CD62P+) and activated endothelial-derived EVs (CD62E+) and (e) tissue factor (CD142+). (PDF) [file pone.0309112.s002.pdf]

**S2 Fig. Flowcytometry setup and gating strategy for characterization of EV subpopulations.** (a) Calibration for determination of EVs in the FITC vs. SSC dot plot by using 0.16–0.5 µm standard Megamix SSC-Plus beads; Approx. 90% of the counts in the gate were detected within the EV size-gate, as described in Taxiarchis et al, 2023. Events positive to Annexin V were considered as EVs. Process of choosing the subpopulations of: (b) MASP2+, TCC+, MPO+, TCC+MPO+, MASP2+MPO+ EVs. (c) CFD+, C3a+, CFD+MPO+, C3a+MPO+ EVs. (d) endothelial-derived EVs (CD144+ and CD54+), activated platelet-derived EVs (CD62P+) and activated endothelial-derived EVs (CD62E+) and (e) tissue factor (CD142+).

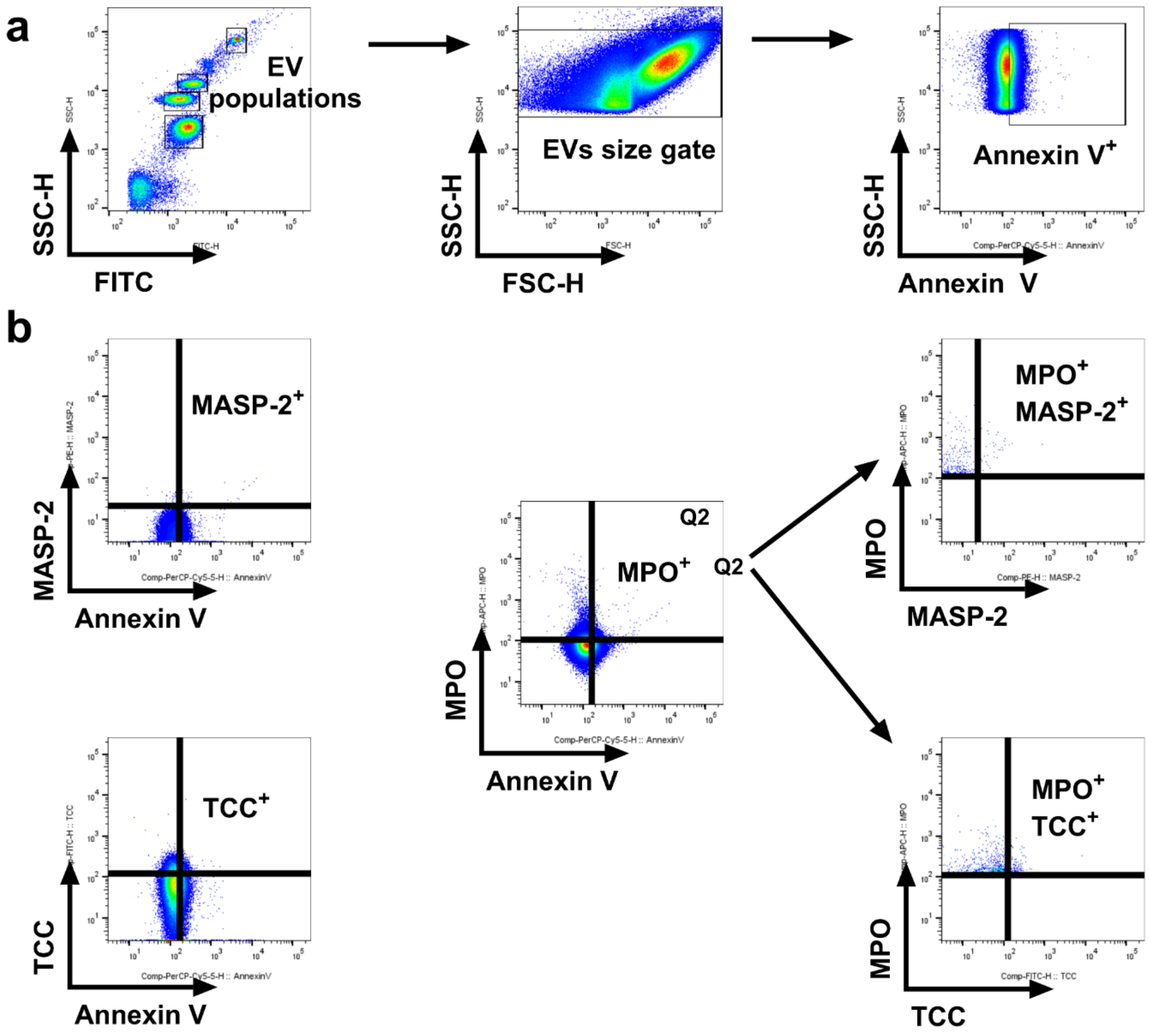

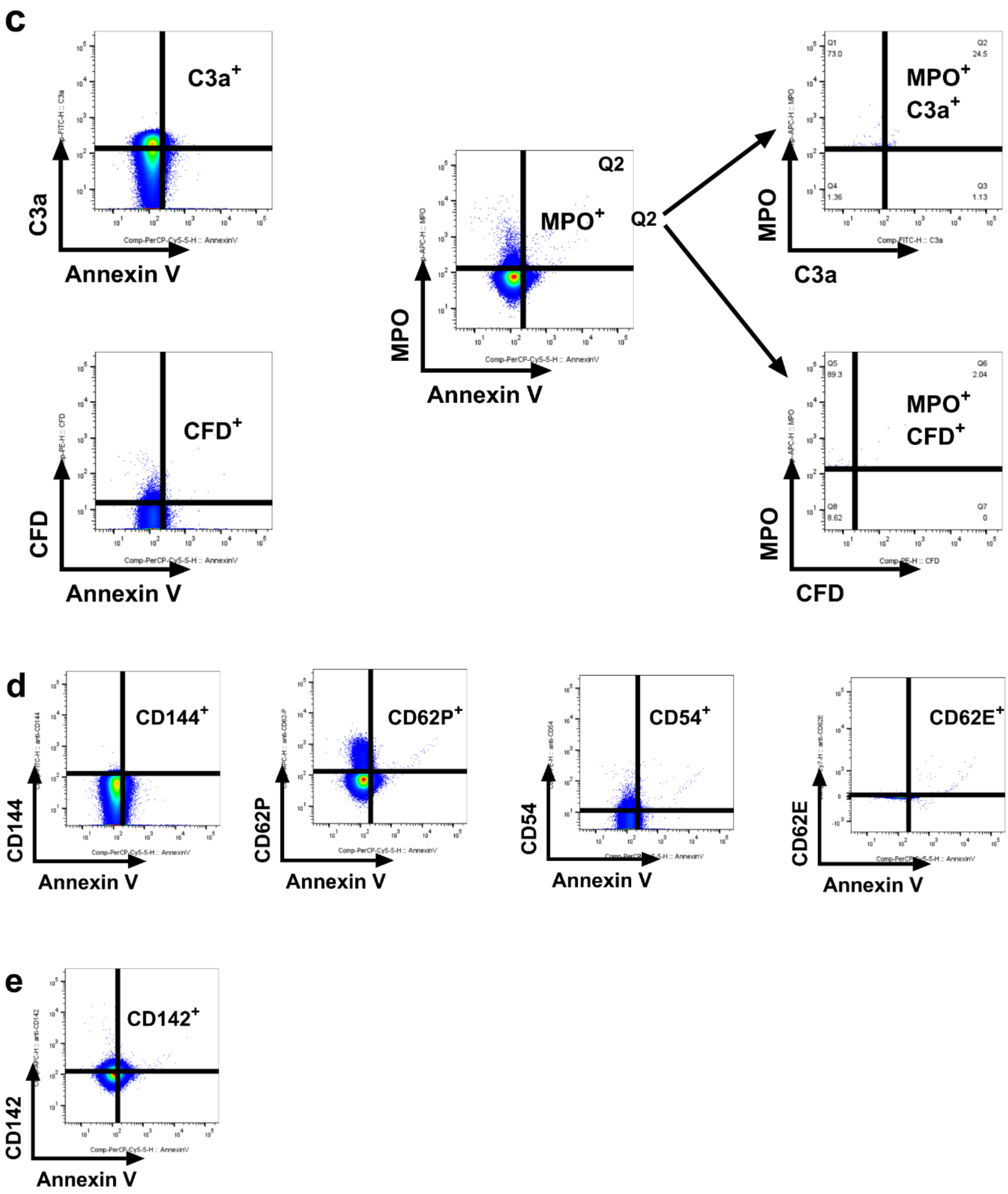
c
C3a+
C3a
Annexin V
CFD+
CFD
Annexin V
Q2
MPO+
MPO
Annexin V
Q2
Q1
73.0
Q2
24.5
MPO+
C3a+
Q4
1.36
Q3
1.13
MPO
C3a
Q5
89.3
Q6
2.04
MPO+
CFD+
Q8
8.62
Q7
0
MPO
CFD
d
CD144+
CD144
Annexin V
CD62P+
CD62P
Annexin V
CD54+
CD54
Annexin V
CD62E+
CD62E
Annexin V
e
CD142+
CD142
Annexin V
