# Supplementary material for: The role of complement and extracellular vesicles in the development of pulmonary embolism in severe COVID-19 cases
Source: PLoS One. 2024 Aug 23;19(8):e0309112. doi: 10.1371/journal.pone.0309112 (PMC11343408; doi:10.1371/journal.pone.0309112)

S3 Fig. Fluorescence minus one (FMO) control. To identify all our sub-populations, all gates were controlled in pooled plasma derived from the patients using a stain that lacks just one of the fluorescent markers of interest.

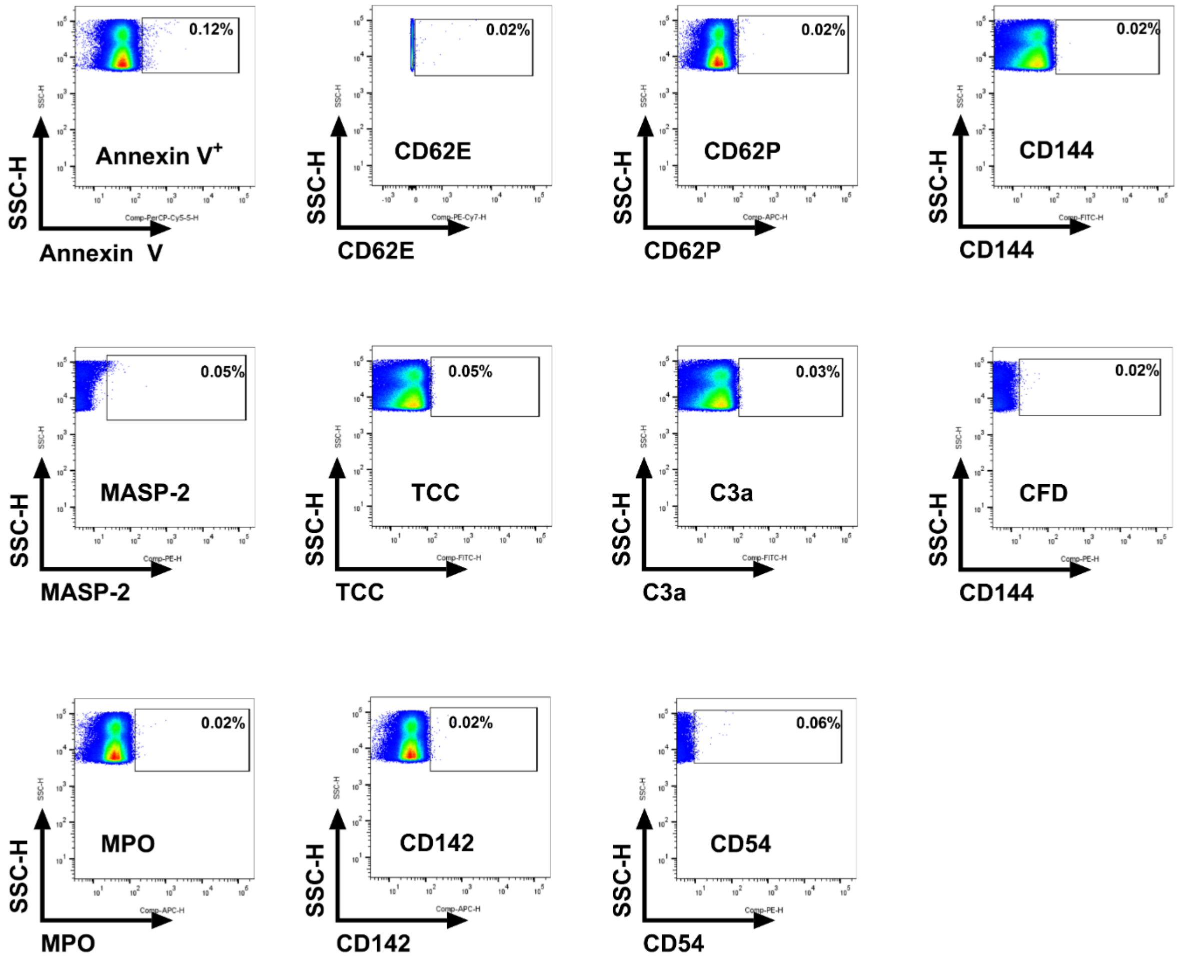

Supplement: S2 Fig — To identify all our sub-populations, all gates were controlled in pooled plasma derived from the patients using a stain that lacks just one of the fluorescent markers of interest. (PDF) [file pone.0309112.s003.pdf]
