# Supplementary material for: The role of complement and extracellular vesicles in the development of pulmonary embolism in severe COVID-19 cases
Source: PLoS One. 2024 Aug 23;19(8):e0309112. doi: 10.1371/journal.pone.0309112 (PMC11343408; doi:10.1371/journal.pone.0309112)

S4 Fig. Extracellular vesicles (EVs). The outcomes are given as events/µL. The Mann-Whitney test was used for comparisons, and the corresponding p-value is displayed in the upper right corner of the image. (no PE= no pulmonary embolism subgroup; PE= pulmonary embolism subgroup).

Complement carrying EVs

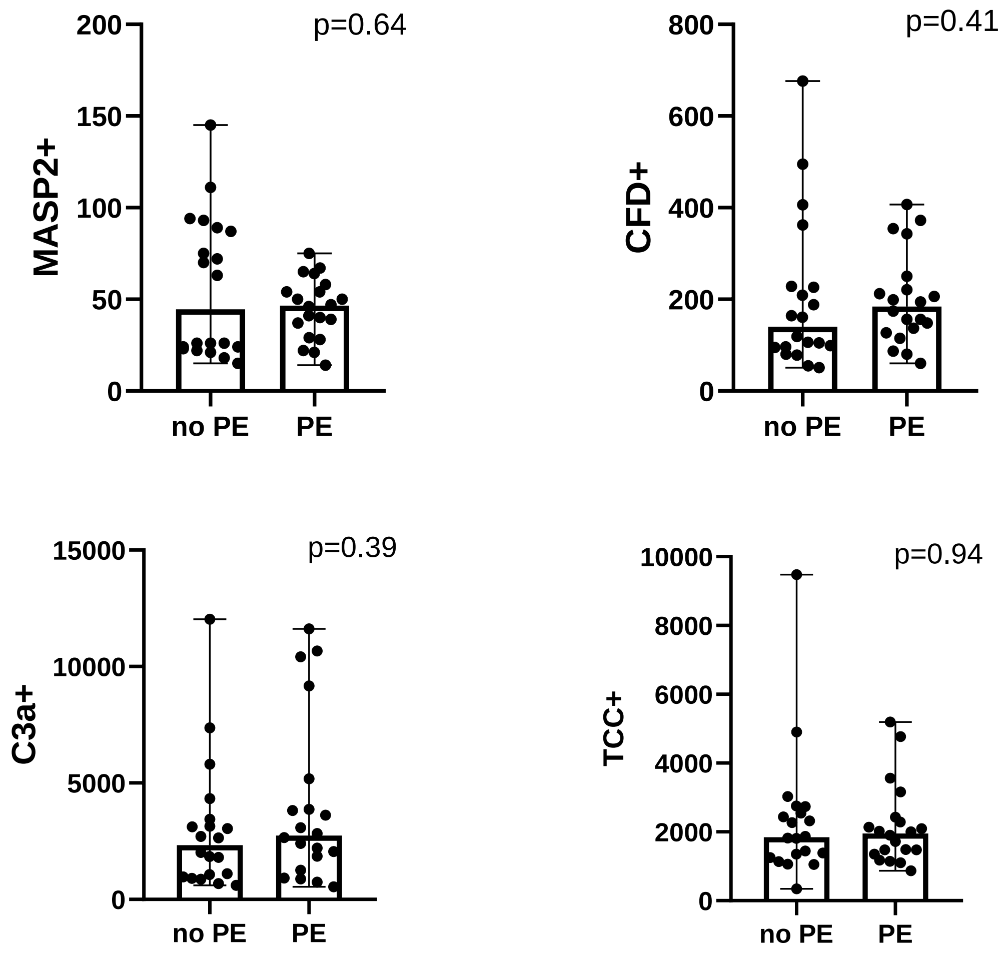

TF carrying EVs

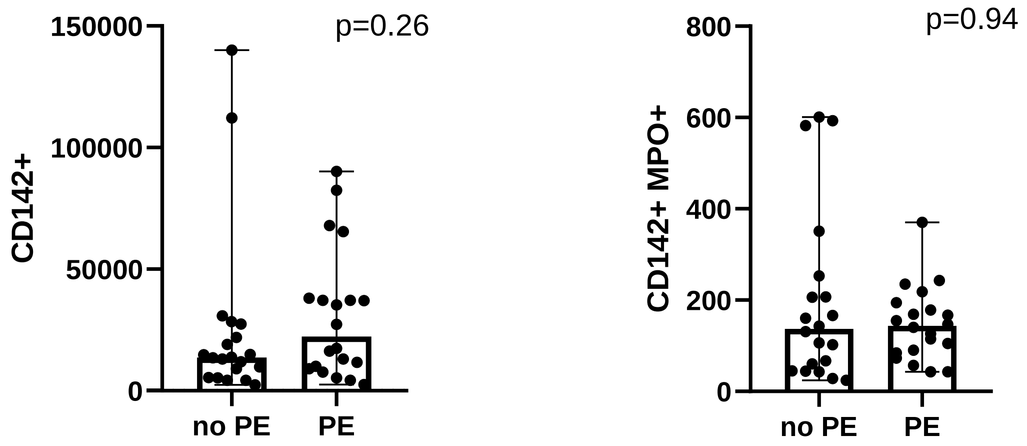

Neutrophil-derived EVs

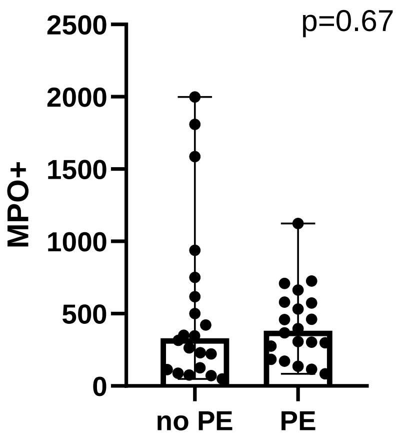

Complement carrying, neutrophil-derived EVs

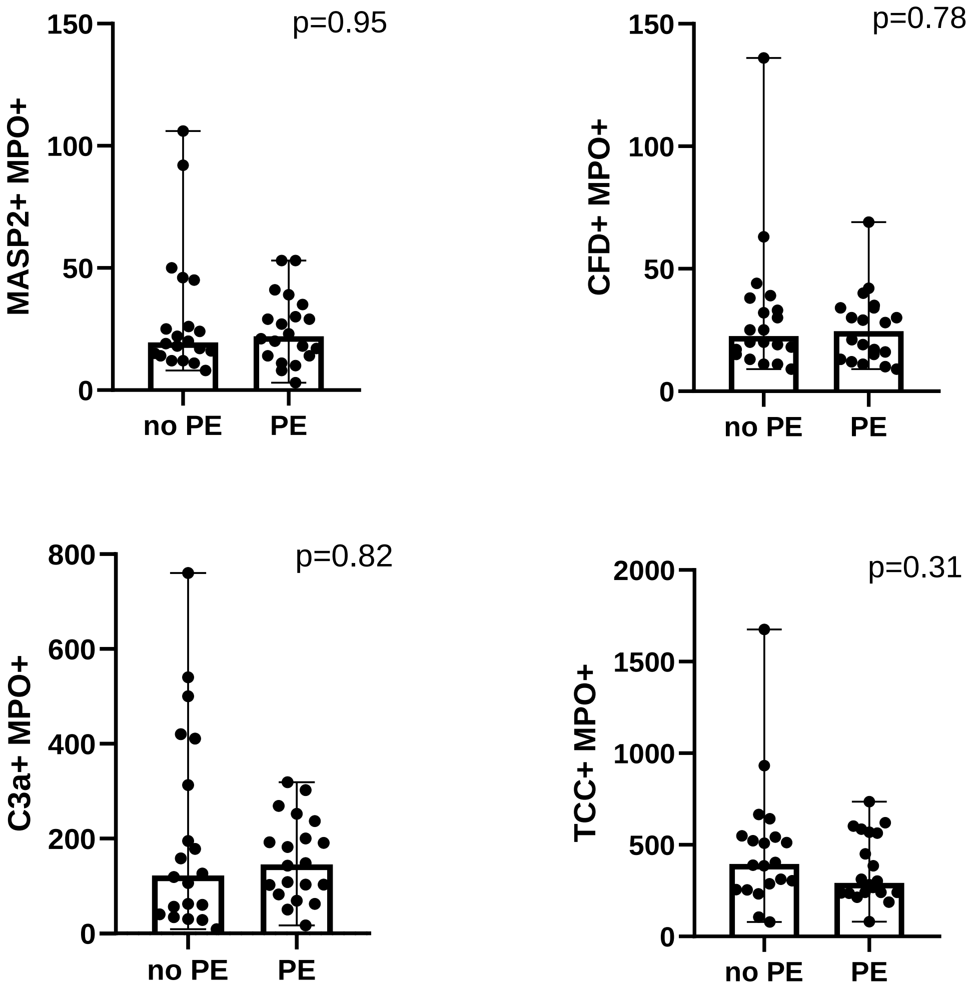

Endothelium-derived EVs

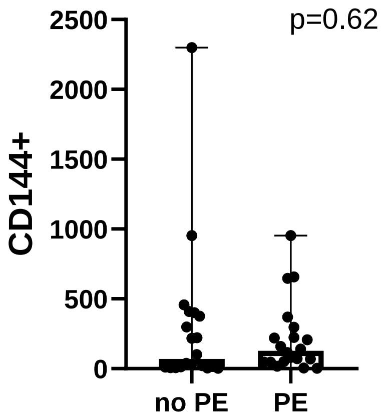

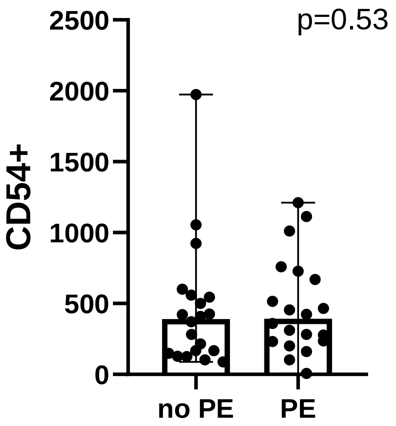

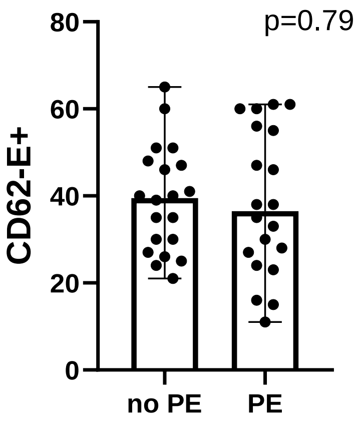

Platelets-derived EVs

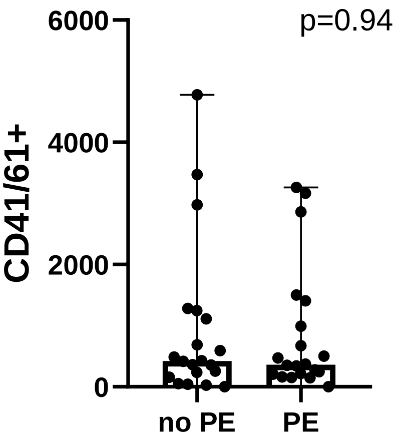

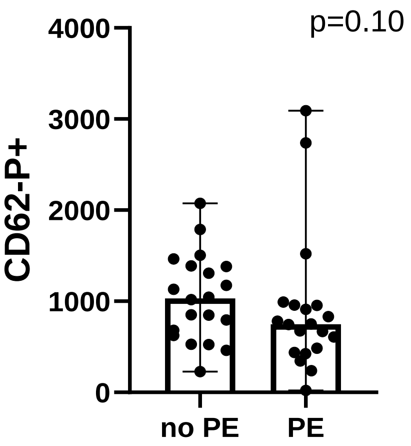

Supplement: S3 Fig — The outcomes are given as events/μL. The Mann-Whitney test was used for comparisons, and the corresponding p-value is displayed in the upper right corner of the image. (no PE = no pulmonary embolism subgroup; PE = pulmonary embolism subgroup). (PDF) [file pone.0309112.s004.pdf]
