# Supplementary material for: The role of complement and extracellular vesicles in the development of pulmonary embolism in severe COVID-19 cases
Source: PLoS One. 2024 Aug 23;19(8):e0309112. doi: 10.1371/journal.pone.0309112 (PMC11343408; doi:10.1371/journal.pone.0309112)

S5 Fig. Tissue factor carrying extracellular vesicles (CD 142+) in all patients (panel A) and in all patients' whiteout 2 outstanding outliers in the subgroup without pulmonary embolism (Panel B). The outcomes are given as events/µL. The Mann-Whitney test was used for comparisons, and the corresponding p-value is displayed in the upper right corner of the image. (no PE= no pulmonary embolism subgroup; PE= pulmonary embolism subgroup)

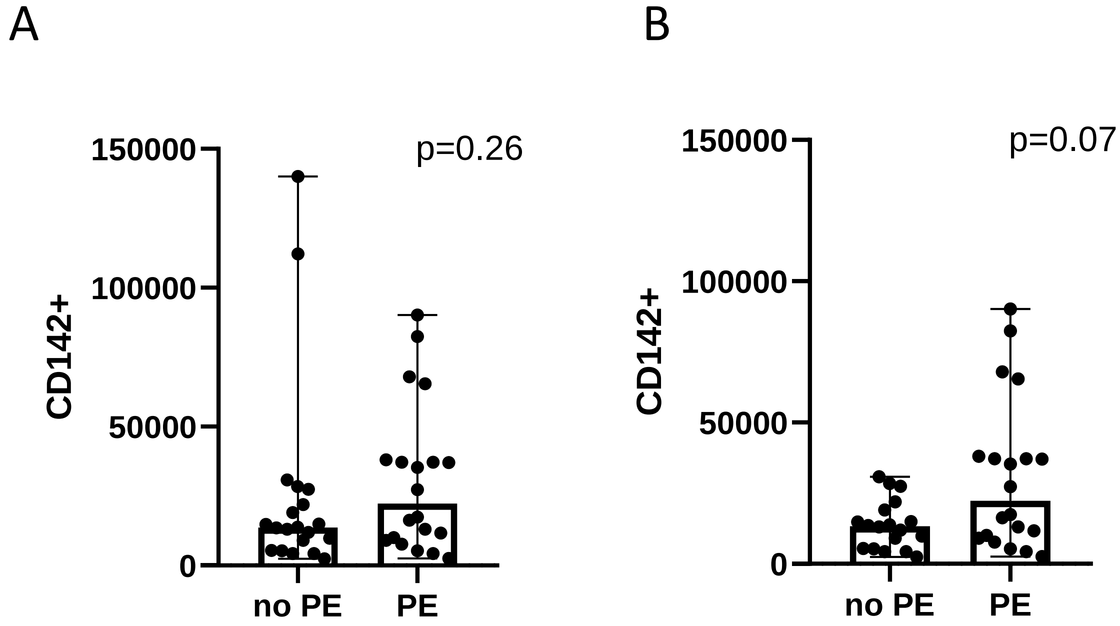

Supplement: S4 Fig — Tissue factor carrying extracellular vesicles (CD 142+) in all patients (panel A) and in all patients’ whiteout 2 outstanding outliers in the subgroup without pulmonary embolism (Panel B). The outcomes are given as events/μL. The Mann-Whitney test was used for comparisons, and the corresponding p-value is displayed in the upper right corner of the image. (no PE = no pulmonary embolism subgroup; PE = pulmonary embolism subgroup). (PDF) [file pone.0309112.s005.pdf]
