# Supplementary figures and images for: The role of complement and extracellular vesicles in the development of pulmonary embolism in severe COVID-19 cases
Source: PLoS One. 2024 Aug 23;19(8):e0309112. doi: 10.1371/journal.pone.0309112 (PMC11343408; doi:10.1371/journal.pone.0309112)

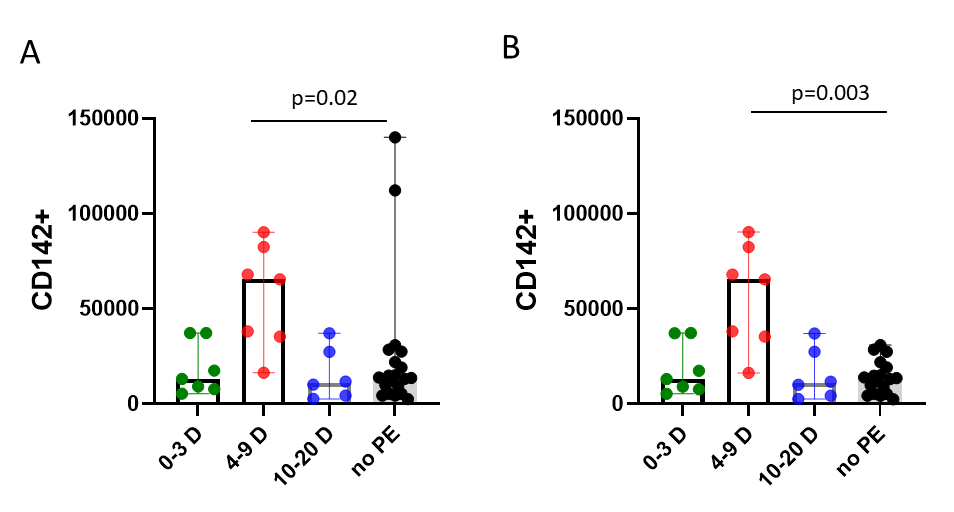

Supplement: S5 Fig — Tissue factor carrying extracellular vesicles (CD 142+) in patient subgroups related to the occurrence of PE: early (0–3 days), interim (4–9 days), and late (10–20 days), and in the subgroup without pulmonary embolism including (panel A) and excluding (panel B) two outstanding outliers. The outcomes are given as events/μL The Mann-Whitney test was used to compare the interim subgroup (4–9 days) with the subgroup without pulmonary embolism, and the corresponding p-value is displayed on the top of the image. (D = day of ICU stay; no PE = no pulmonary embolism subgroup) (TIF) [file pone.0309112.s006.tif]
